# Flow cytometric resolution of yeast is affected by enzymatic treatment and culture media

**DOI:** 10.1002/2211-5463.13456

**Published:** 2022-08-02

**Authors:** Mei Wang, Feizhen Wu, Zhongkai Gu

**Affiliations:** ^1^ Department of Geriatrics, Shanghai General Hospital Shanghai Jiao Tong University School of Medicine Shanghai China; ^2^ Laboratory of Epigenetics at Institutes of Biomedical Sciences, and Intelligent Medical Institute Fudan University Shanghai China; ^3^ Key Laboratory of Birth Defects Children's Hospital of Fudan University Shanghai China

**Keywords:** cell cycle, cytometric resolution, flow cytometry, *Saccharomyces cerevisiae*, SYBR Green I, yeast

## Abstract

*Saccharomyces cerevisiae* is an important model organism and a typical fungal representative for studies of eukaryotes. The cell cycle of yeast can be analyzed by flow cytometry, and refining the cytometric resolution of the cell cycle with this technique is important. Such refinement is potentially influenced by multiple factors, including enzymatic treatment and variations in the culture media used, although this has been subject to only limited investigation. Here, we examined the effect of different enzymatic pre‐treatments and various media on cytometric resolution. We show that cytometric resolution is significantly altered by both enzymatic conditions and the media used. Culture media with different amino nitrogen concentrations potentially impact the protein content in the yeast cell wall, which may affect the permeability of the cell wall and alter cytometric resolution. The present study provides beneficial technical information about the influence of media and enzymes on the cytometric resolution of the yeast cell cycle and most likely other fungi, which should be considered in future research.

## Graphical abstract

The cytometric resolution of the cell cycle of yeast as measured by flow cytometry is influenced by both enzymatic conditions and the culture medium used. This phenomenon may be a result of variations in the protein ratio in the cell wall, and this fluctuation in cytometric resolution should be considered for future studies.

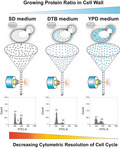

**Abbreviations**
CV: coefficient of variation
DTB: dextrose‐tryptone‐broth
SD: synthetic defined
YPD: yeast extract‐peptone‐dextrose

Yeast is a type of eukaryotic unicellular organism widely used for fermentation in the wine industry [1]. It is also typically utilized as a model organism, especially for the purpose of genetic research, as a result of its simple medium requirements, controllable cultivation conditions, short lifespan, and relatively well‐understood genome [2, 3, 4]. Yeast is a popular system for investigating cell growth and division, and flow cytometry is the most common approach for assessing the cell cycle in yeast, which requires enzymes for sample pre‐treatment prior to cytometric detection to refine the cytometric resolution of the yeast cell cycle [5]. However, the duration of enzymatic digestion possibly influences the resolution, and studies regarding this are limited. In addition, yeast cells are capable of growing in various media, from the minimal medium to yeast extract‐peptone‐dextrose (YPD) medium [6]; besides, little is known regarding the influence of medium alteration on the cytometric resolution of the yeast cell cycle.

As indicated by some early findings in the present study, both an altered duration of enzymatic exposure and various yeast media appeared to affect the reproducibility of the cytometry results. Therefore, to better understand the potential influence, an assay matrix composed of multiple enzymatic conditions was simultaneously applied to yeast cells cultivated in various media. As a result, when the digestive duration of RNase A and Proteinase K was extended from 1 to 24 h, the coefficient of variation (CV) values of the G1 and G2 phases were reduced, reflecting the separated and sharpened histogram peaks of the two phases, and yielding an elevated cytometric resolution of the yeast cell cycle [7]. Furthermore, the alterations in the resolution became increasingly significant when the growth medium was switched from the minimal medium to synthetic defined (SD) medium [6], dextrose‐tryptone‐broth (DTB) medium [8] and YPD medium. Additionally, Proteinase K was confirmed to be more responsible for the elevated resolution than RNase A because the elevation was more sensitive to the extension of Proteinase K treatment. Moreover, the sensitivity of the resolution to Proteinase K became remarkable when the medium was switched, which was initially suspected to be the result of an altered permeability of cell wall caused by the increasing proportion of the amino nitrogen in the medium.

The present study highlights the relationship between culture medium alteration, the duration of enzymatic treatment, and the cytometric resolution of the cell cycle, demonstrating variations in the resolution when enzymatic treatment time and medium were altered. In addition, the variations of the resolution were suspected to be attributed to the varied amino nitrogen proportion in the different media, leading to various protein contents and a diverse permeability of the yeast cell wall. Therefore, the present study provides beneficial technical details regarding the multiple influences on the cytometric resolution of the yeast cell cycle and possibly that of other fungi, which should be considered in the design of future studies.

## Materials and methods

### Cell cultivation

*Saccharomyces cerevisiae* S288C (MATa *his3*Δ1 *leu2*Δ0 *met15*Δ0 *ura3*Δ0) [9, 10] strain was grown at 30 °C in minimal medium (Table S1), SD medium (Table S2) [6], DTB medium (Table S3) [8], and YPD medium (Table S4) [6]. Cells were collected when OD_595 nm_ of 1.0 was reached [11, 12, 13].

### Cell fixation

Cells were collected when the cell density of each sample was 2.75 × 10^8^ cells·mL^−1^. The cells, together with fresh medium (1 mL), were transferred into a centrifuge tube and centrifuged at 20 000 rcf for 3 min, followed by supernatant removal. The cell pellet was resuspended in ddH_2_O (1 mL) by pipetting and then centrifuged again at 20 000 rcf for 3 min. The supernatant was discarded, and the cell pellet was resuspended in 1 mL of 75% ethanol at 4 °C (catalog. no. 10009218; Shanghai Reagent Co. Ltd, Shanghai, China) for fixation. As a result of concerns about instrument maintenance, such that any living yeast cell would possibly contaminate the flow cytometer, samples were fixed for 1 week prior to being loaded onto the machine. Samples were stored at 4 °C until the assay was performed.

### Cell resuspension

Samples in centrifuge tubes were retrieved from storage in the refrigerator and centrifuged at 20 000 rcf for 1 min, followed by supernatant removal. The cells were resuspended in ddH_2_O (1 mL) by pipetting and then centrifuged at 20 000 rcf for 2 min. The supernatant was then discarded, and the cell pellet was resuspended in 50 mm sodium citrate (1.5 mL; catalog. no. 10019408; Shanghai Reagent Co. Ltd). The suspension was then divided into three aliquots, each of which contained a volume of 500 μL.

### Cell staining and flow cytometry detection

The staining method used in the present study was performed as described previously [14], but with modifications. In each sample aliquot of the three above, 20 mg·mL^−1^ RNase A (10 μL; catalog. no. R4875, dissolved in ddH_2_O; Sigma‐Aldrich, St Louis, MO, USA) was added, and then the samples were incubated at 37 °C in a mixing incubator (ThermoMixer^®^; Eppendorf, Hamburg, Germany) at 950 r.p.m. for 1, 6 or 24 h. After incubation, the cells were centrifuged at 20 000 rcf for 3 min, followed by supernatant removal. The cells were then resuspended in 50 mm sodium citrate (500 μL). The resuspended cells were then store at 4 °C until all the samples were ready for the Proteinase K assay.

Subsequently, 20 mg·mL^−1^ Proteinase K (10 μL; catalog. no. P6556; Sigma‐Aldrich) was added to each sample, and the samples were then incubated in the mixing incubator at 50 °C for 0, 1, 3, 6 or 24 h. At each timepoint, 100 μL of liquid containing yeast cells was collected from the sample tube and the Proteinase K was removed by centrifuging the sample at 20 000 rcf for 3 min. The cell pellet was then resuspended in 50 mm sodium citrate (500 μL) and stored at 4 °C until all the samples were ready for the staining assay.

Once all the samples were ready, they were all retrieved from the refrigerator simultaneously. Each sample received Triton X‐100 (1.25 μL; catalog. no. T8787; Sigma‐Aldrich) and 10 000 × SYBR Green I (0.1 μL; catalog. no. S7563; Thermo Fisher Scientific, Waltham, MA, USA) and was thoroughly vortexed. The staining was performed on all the samples on a rotating mixer (HulaMixer; catalog. no. 15920D; Thermo Fisher Scientific) at 4 °C for 24 h. Sonication was not suggested because the refinement of cytometric resolution was found very limited when an ultrasonic tank (Type 2201B; Shanghai Shengyan Ultrasonic Co. Ltd, Shanghai, China) was used at 4 °C for 5 min (Fig. S1A,B,D,E,G,H,J,K). All the samples were simultaneously loaded onto a LSRFortessa flow cytometer (Becton‐Dickinson, Franklin Lakes, NJ, USA). The signals of 20 000 cells were detected using fascdiva, version 6.2 (Becton‐Dickinson). The unstained controls confirmed a low autofluorescence background of the cells grown in any medium (Fig. S1C,F,I,L).

### Voltage fixation

During signal collection on the flow cytometer, the voltage was fixed and applied to all the samples to evaluate the samples in parallel. In detail, the voltages were set to FSC‐A: 500, SSC‐A: 364, SSC‐H: 364, SSC‐W: 364, and FITC‐A: 460.

### Data analysis: gating

To illustrate the exact cytometry of each sample, no gate was constructed to prevent signal selection. Otherwise, all signals were included to determine whether cell fragments were largely generated during the assay. Alternatively, a quad gate was set for each pseudocolor dot plot to display the contraction of the signals during the refinement of the resolution. The dot plots of yeast cells growing in the same medium shared the same setting of the quad gate. Data visualization and analysis were achieved via flowjo, version 7.6.1 (FlowJo LLC, Ashland, OR, USA). Manual line gatings were applied to histograms to initially display the cell cycles.

### Data analysis: automatic cell cycle statistics

Following the histogram displays, automatic cell cycle statistics were performed on each sample with flowjo. The analysis algorithm used was Dean–Jett–Fox, whereas the other options were set to default. No additional settings were used.

## Results

### Cytometric resolution of the yeast cell cycle was refined when the enzymatic exposure was extended, but the refinement was altered when the medium was switched

**Fig. 1:**
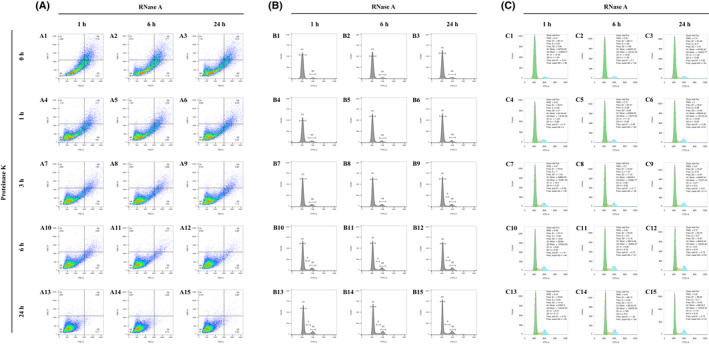
The flow cytometry matrix results of yeast cells grown in the minimal medium and treated with an RNase A digestion time course and a Proteinase K digestion time course. (A) Pseudocolor dot plots of all the flow cytometry results. The horizontal axis is FSC‐A and the vertical one is SSC‐A. A quad gate was set to 150 K for FSC‐A and 110 K for SSC‐A. (B) Histograms of all the flow cytometry results, which are matched to the previous dot plots by letter and number [e.g. (B1) to (A1), (B2) to (A2), (B3) to (A3)]. The maximum of the vertical axes is fixed to 2.0 K and the maximum of the horizontal axes is fixed to 125 K. Manual line gatings are performed on the histograms to display the separated peaks of different cell cycle phases. ‘G1’ means the G1 phase, ‘G2’ means the G2 phase, and ‘S' means the synthesis phase. (C) Automatic cell cycle statistics of the flow cytometry results, which are matched to the previous histograms and dot plots by letter and number [e.g. (C1) to (B1) to (A1), (C2) to (B2) to (A2), (C3) to (B3) to (A3)]. The green color represents the automatically analyzed G1 phase frequencies. The blue color represents the G2 phase frequencies, and the yellow color represents the S phase frequencies. As a result of limitations of the flowjo software, the maximum of the vertical axes is non‐adjustable and is unable to match that of the previous histograms, which are all fixed to 2.0 K. [Colour figure can be viewed at wileyonlinelibrary.com]

The minimal medium was first utilized as a control medium, and the yeast cells growing in the minimal medium yielded a high resolution even when the enzymes were only briefly employed (1 h for RNase A, 0 h for Proteinase K), which was supported by the separated peaks of the G1 and G2 phases (Fig. 1B1) in the histogram. However, despite the dot signals being quickly concentrated in the Q4 square (Fig. 1A1–A15), the two peaks illustrated a restricted improvement by the limited sharpening when the duration of enzymatic exposure of both two enzymes was extended to 24 h (Fig. 1B1–B15). The automatic cell cycle statistics also supported the limitation above because the CV value of the G1 peak only shrank by 6.31, and the G2 peak CV value only shrank by 3.76 (G1 CV: 13.46–7.15 = 6.31; G2 CV: 11.94–8.18 = 3.76) (Fig. 1C1,C15). This result implied that the cell wall of yeast grown in the minimal medium had been highly permeable at the beginning. Therefore, little space was left for refining the resolution by extended enzymatic exposure in the minimal medium.

**Fig. 2:**
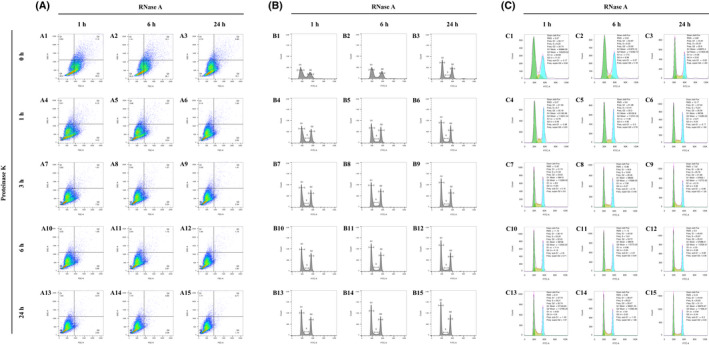
The flow cytometry matrix results of yeast cells grown in SD medium and treated with an RNase A digestion time course and a Proteinase K digestion time course. (A) Pseudocolor dot plots of all the flow cytometry results. The horizontal axis is FSC‐A and the vertical one is SSC‐A. A quad gate was set to 90 K for FSC‐A and 110 K for SSC‐A. (B) Histograms of all the flow cytometry results, which are matched to the previous dot plots by letter and number [e.g. (B1) to (A1), (B2) to (A2), (B3) to (A3)]. The maximum of the vertical axes is fixed to 2.0 K and the maximum of the horizontal axes is fixed to 125 K. Manual line gatings are performed on the histograms to display the separated peaks of different cell cycle phases. ‘G1’ means the G1 phase, ‘G2’ means the G2 phase, and ‘S' means the synthesis phase. (C) Automatic cell cycle statistics of the flow cytometry results, which are matched to the previous histograms and dot plots by letter and number [e.g. (C1) to (B1) to (A1), (C2) to (B2) to (A2), (C3) to (B3) to (A3)]. The green color represents the automatically analyzed G1 phase frequencies. The blue color represents the G2 phase frequencies, and the yellow color represents the S phase frequencies. As a result of limitations of the flowjo software, the maximum of the vertical axes is non‐adjustable and is unable to match that of the previous histograms, which are all fixed to 2.0 K. [Colour figure can be viewed at wileyonlinelibrary.com]

When grown in SD medium, yeast cells initially yielded a lower cytometric resolution, as the separated G1 and G2 peaks were lower and wider (Fig. 2B1) compared to the peaks in the minimal medium (Fig. 1B1). However, the extended RNase A and Proteinase K treatments (from 1 to 24 h; Fig. 2B1–B15) elevated the resolution by sharpening the two peaks and concentrated the signals into the Q4 quarter (Fig. 2A1–A15). This phenomenon was furtherly supported by an obvious decrease in the CV values of the G1 and G2 phases (G1 CV: 20.83–5.59 = 15.24; G2 CV: 11.51–3.44 = 8.07; Fig. 2B1,B15), which was the result of the extended enzymatic digestion. Furthermore, the obviously decreased CV values suggested that the extension of RNase A and Proteinase K treatments lifted the cytometric resolution of the cell cycle of yeast grown in SD medium more significantly than the one in the minimal medium, implying that the dye permeated the yeast cell wall more difficultly in SD medium as compared to the minimal medium until the enzymatic digestions promoted the permeability.

**Fig. 3:**
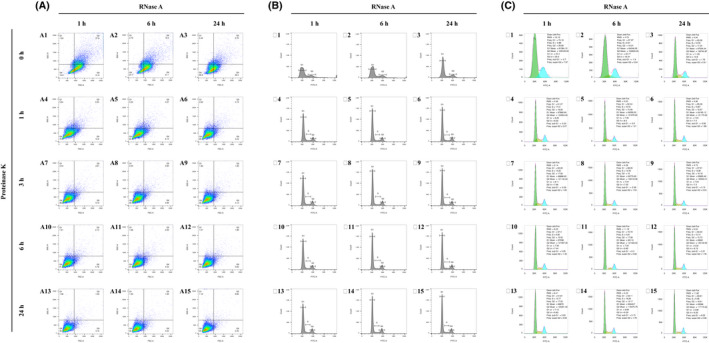
The flow cytometry matrix results of yeast cells grown in DTB medium and treated with an RNase A digestion time course and a Proteinase K digestion time course. (A) Pseudocolor dot plots of all the flow cytometry results. The horizontal axis is FSC‐A and the vertical one is SSC‐A. A quad gate was set to 90 K for FSC‐A and 80 K for SSC‐A. (B) Histograms of all the flow cytometry results, which are matched to the previous dot plots by letter and number [e.g. (B1) to (A1), (B2) to (A2), (B3) to (A3)]. The maximum of the vertical axes is fixed to 2.0 K and the maximum of the horizontal axes is fixed to 125 K. Manual line gatings are performed on the histograms to display the separated peaks of different cell cycle phases. ‘G1’ means the G1 phase, ‘G2’ means the G2 phase, and ‘S' means the synthesis phase. (C) Automatic cell cycle statistics of the flow cytometry results, which are matched to the previous histograms and dot plots by letter and number [e.g. (C1) to (B1) to (A1), (C2) to (B2) to (A2), (C3) to (B3) to (A3)]. The green color represents the automatically analyzed G1 phase frequencies. The blue color represents the G2 phase frequencies, and the yellow color represents the S phase frequencies. As a result of limitations of the flowjo software, the maximum of the vertical axes is non‐adjustable and unable to match that of the previous histograms, which are all fixed to 2.0 K. [Colour figure can be viewed at wileyonlinelibrary.com]

In addition, yeast cells grown in DTB medium initially yielded a much lower resolution as compared to the one in SD medium, as the peaks of the G1 and G2 phases tended to be stuck to each other when cells were treated with 1 h of RNase A but without Proteinase K treatment (Fig. 3B1). Extended treatments of both enzymes, however, successfully separated the G1 and G2 phase peaks by illustrating an obvious S phase (Fig. 3B1–B15) and concentrating the signals into the Q4 square (Fig. 3A1–A15). In detail, RNase A treatment for 24 h elevated the resolution of the two peaks by reducing the CV values (G1 CV: 24.4–5.96 = 18.44; G2 CV: 22.3–6.05 = 16.25; Fig. 3C1,C15). Moreover, Proteinase K treatment also largely contributed to the elevation above, especially when RNase A treatment was fixed but Proteinase K digestion was extended. Therefore, the decreases of the two CV values in DTB medium, compared to those in SD medium, suggested that the two enzymes more significantly elevated the cytometric resolution of the cell cycle. This result also implied that the yeast cells were preliminarily less permeable to the dye in DTB medium, whereas increasing the duration of enzymatic digestion to 24 h obviously lifted the permeability of the yeast cells.

**Fig. 4:**
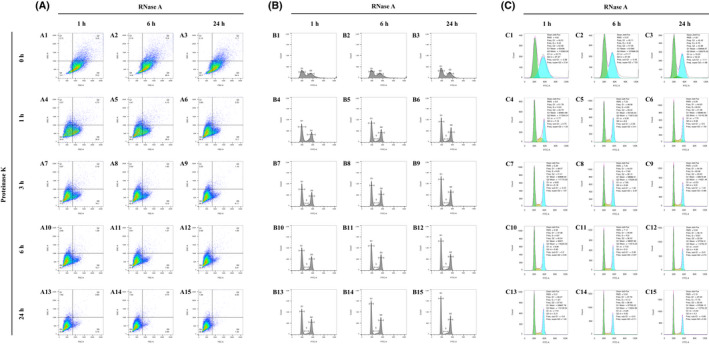
The flow cytometry matrix results of yeast cells grown in YPD medium and treated with an RNase A digestion time course and a Proteinase K digestion time course. (A) Pseudocolor dot plots of all the flow cytometry results. The horizontal axis is FSC‐A and the vertical one is SSC‐A. a quad gate was set to 80 K for FSC‐A and 100 K for SSC‐A. (B) Histograms of all the flow cytometry results, which are matched to the previous dot plots by letter and number [e.g. (B1) to (A1), (B2) to (A2), (B3) to (A3)]. The maximum of the vertical axes is fixed to 2.0 K and the maximum of the horizontal axes is fixed to 125 K. Manual line gatings are performed on the histograms to display the separated peaks of different cell cycle phases. ‘G1’ means the G1 phase, ‘G2’ means the G2 phase, and ‘S’ means the synthesis phase. (C) Automatic cell cycle statistics of the flow cytometry results, which are matched to the previous histograms and dot plots by letter and number [e.g. (C1) to (B1) to (A1), (C2) to (B2) to (A2), (C3) to (B3) to (A3)]. The green color represents the automatically analyzed G1 phase frequencies. The blue color represents the G2 phase frequencies, and the yellow color represents the S phase frequencies. As a result of limitations of the flowjo software, the maximum of the vertical axes is non‐adjustable and unable to match that of the previous histograms, which are all fixed to 2.0 K. [Colour figure can be viewed at wileyonlinelibrary.com]

Finally, yeast cells grown in YPD medium initially yielded a low resolution similar to that seen with DTB medium, with low and wide G1 and G2 phase peaks stuck to each other, even after RNase A treatment for 24 h without Proteinase K treatment (Fig. 4B3). However, the resolution was largely optimized by the Proteinase K treatment for 1 h, which immediately sharpened the appearance of the two peaks (Fig. 4B4–B6). The optimization was also confirmed by statistics, which showed that the two CV values quickly jumped by 10.96 and 20.25 (G1 CV: 22.73–11.77 = 10.96; G2 CV: 27.37–7.12 = 20.25; Fig. 4C1,C4). In addition, when the enzymatic digestion for both RNase A and Proteinase K was extended to 24 h, there was a gradual increase in the S phase, and there was a continued sharpening of the two peaks, which contributed to a significantly optimized resolution (Fig. 4B1–B15). The statistics also confirmed the optimization of the CV values, which dropped to 5.22 and 4.3 (Fig. 4C15) and were much lower than the initial values (G1 CV: 22.73, G2 CV: 27.37; Fig. 4C1). Furthermore, the decrease in the G2 CV value in YPD medium, which was reduced by 23.07 (G2 CV: 27.37–4.3 = 23.07; Fig. 4C1,C15), was the most significant among the four media. The signals were rapidly concentrated in the Q4 quarter (Fig. 4A1–A15), which also supported the phenomenon. All of the statistics suggested that, initially, yeast cells growing in YPD were minimally permeable with a low resolution, although the resolution was significantly elevated as compared to other media after enzymatic digestion for 24 h.

In summary, disparities in the refinement of the G1 and G2 values in yeast cells grown in different media under the different enzymatic conditions implied that RNase A and Proteinase K remarkably refined the cytometric resolution with extended enzymatic exposure. However, the permeability of yeast cells probably varied when grown in various media, which resulted in a fluctuant refinement of the cytometric resolution of the yeast cell cycle.

## Discussion

### The CV value statistics indicated that yeast grown in various growth media caused different permeabilities of cells

**Fig. 5:**
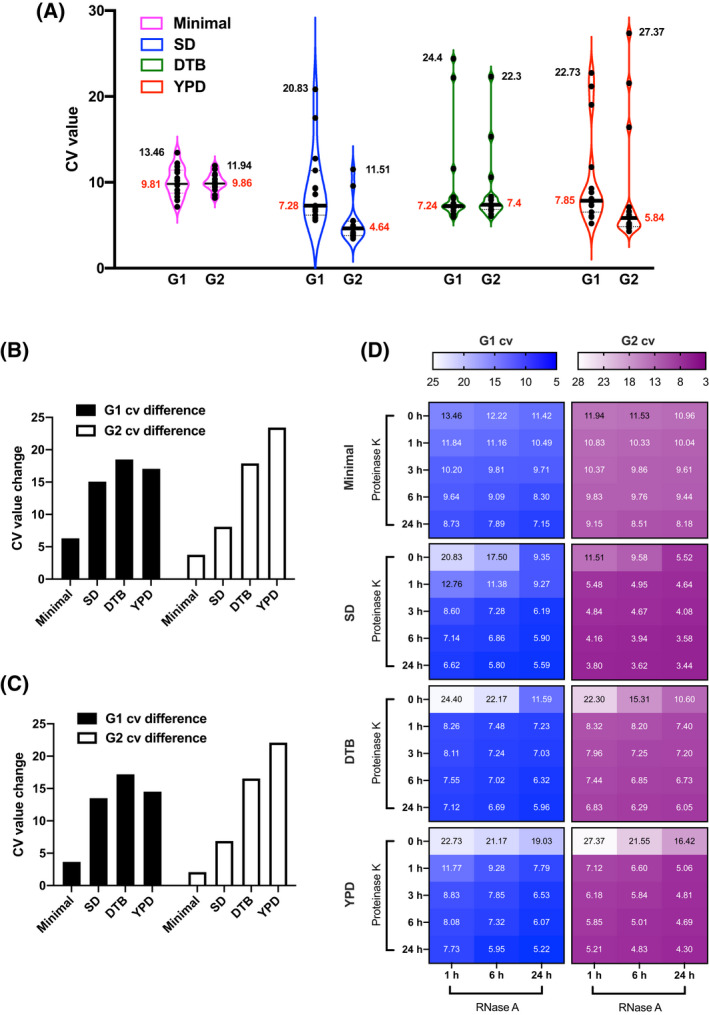
The statistics of the CV values of the G1 and G2 phases of yeast cells grown in the four types of growth media. (A) a violin plot of the statistics. The statistics are grouped into the G1 and G2 phases. The purple violins show the statistics of the two phases of yeast cells grown in the minimal medium. The blue violins show the statistics of the two phases of yeast cells grown in SD medium. The green violins show the statistics of the two phases of yeast cells grown in DTB medium. The red violins show the statistics of the two phases of yeast cells grown in YPD medium. The black number is the maximal CV value among the counted values in each phase of the cell sample grown in each medium, whereas the red number is the median one among the values. (B) A bar plot of the CV value differences between the maximal values and the minimal ones. Black bars represent the CV value differences of the G1 phases in various media. White bars represent the CV value differences of the G2 phases in various media. (C) A bar plot of the CV value differences between the maximal values and the median ones. Black bars represent the CV value differences of the G1 phases in various media. White bars represent the CV value differences of the G2 phases in various media. (D) A heatmap of all the CV values, which are grouped according to the G1 and G2 phases and the four types of media after various RNase a and Proteinase K treatment conditions. The blue color represents the CV values of the G1 phases in the four types of growth media, and the wine‐red color represents the CV values of the G2 phases in the four types of growth media. The legend bars show the upper and lower limits of the ranges of the CV values. [Colour figure can be viewed at wileyonlinelibrary.com]

To evaluate the resolution profile of the yeast cell cycle subjected to different enzymatic conditions, statistical analyses were performed on all of the CV values. When yeast cells were grown in the minimal medium, the fluctuation of the two CV values was limited to relatively narrow ranges (Fig. 5A, purple violins), whereas the ranges became markedly expanded when cells were grown in SD, DTB, and YPD media (Fig. 5A, blue, green, red violins). The maximum‐minimum value difference of the G1 CV value confirmed the range expansion, with an increasing trend being visible when the medium was switched from the minimal to YPD (Fig. 5B, black bars). Additionally, the increasing trend of the G1 CV value was also revealed in the maximum–median value difference (Fig. 5C, black bars). Moreover, the increasing trend of the G2 CV value became even more apparent when the maximum–minimum and the maximum–median value differences were calculated (Fig. 5B,C, white bars). With the increasing trend of the value differences, the two decreasing CV values yielded a refined cytometric resolution and a better cell cycle performance. The resolution reflected the efficiency of the dye penetrating the cell wall and staining the nucleus. The statistics therefore illustrated a variation in the permeability of yeast cells when grown in different types of media.

### The alteration of media probably changed the protein content in the yeast cell wall and led to fluctuations in the cell cycle profile

To understand the reason why various media led to a different resolution, we decided to investigate the yeast cell wall. Typically, the cell wall of yeast cells is mainly composed of chitin, a type of polysaccharide that is made of long‐chain polymers of *N*‐acetylglucosamine [15]. Theoretically, this type of component is not sensitive to Proteinase K digestion. However, protein is also an important component of the yeast cell wall [16]. By reviewing the statistics above, the reduced rates of the G1 CV values over time after the RNase A and Proteinase K treatments were found to differ from each other. In detail, initially, the G1 CV value in the minimal medium was gradually reduced with the enzymatic digestion of both enzymes (Fig. 5D, blue blocks). The G1 CV value began to rapidly decrease after Proteinase K treatment compared to that after RNase A when in SD medium, and this trend continued and even became more obvious in DTB and YPD media (Fig. 5D, blue blocks). Moreover, the situation was similar for the G2 phase because the G2 CV value gradually declined with the enzymatic digestion of both enzymes in the minimal medium (Fig. 5D, wine‐red blocks). Otherwise, Proteinase K treatment for 1 h quickly reduced the value, especially in DTB and YPD media, which was more efficient than the RNase A treatment (Fig. 5D, wine‐red blocks). All of the number changes implied that Proteinase K was more effective for refining the cytometric resolution of the cell cycle compared to RNase A. In consideration of the substrates of RNase A and Proteinase K, which are RNA and proteins, the analysis suggested a diverse proportion of proteins in the cell wall. Therefore, differences in the composition of the cell wall of yeast grown in different media may be responsible for the observed variations in the refinement of the cytometric resolution of the yeast cell cycle.

One reason for the potential differences in the cell walls of yeast grown in different cultivation media may be a result of differences in the concentration of amino nitrogen in the various media. It is theorized that yeast grown in the minimal medium would synthesize a cell wall primarily composed of polysaccharides but not proteins because few amino acids in the minimal medium contributed to the formation of cell wall as a nitrogen source. The concentration of amino nitrogen in the minimal medium is very limited (0.0571 g·L^−1^) (Table S1) and therefore, amino nitrogen would be used first for the essential biological activities of yeast but not synthesis of the cell wall. As a result, this potentially leads to the formation of the cell wall that is primarily composed of polysaccharides, which would loosen the cell wall and make it permeable to RNase A and dye but not sensitive to Proteinase K digestion that specifically targets proteins. By contrast, the concentration gradually increased in SD and DTB media: 0.0666 g·L^−1^ (Table S2) and 0.74 g·L^−1^ (Table S3), respectively. Finally, the YPD medium, which provides the complete nutrients and is a sufficient amino nitrogen source with a concentration of 1.25 g·L^−1^ (Table S4), offers yeast cells an opportunity to recruit more proteins in the cell wall compared to the other three media. As a consequence, the proportion of proteins in the cell wall of yeast grown in YPD medium would be largely elevated, which provides Proteinase K with sufficient space to remarkably refine the cytometric resolution and cell cycle performance, reflecting the plummeting of the CV values (Fig. 5D). Naturally, the altered protein proportion was potentially attributed to the varied metabolism of yeast cells when grown in the different media [17], and the absence or presence of a certain compound in media would possibly lead to such an alteration in the resolution [18]. Although other ingredients in the cell wall could cause fluctuant resolution [19], further investigations are needed to determine the exact components that influence the cytometric resolution of the yeast cell cycle.

In summary, the results obtained in the present study indicate that the duration of enzymatic digestion influences the cytometric resolution of the yeast cell cycle, and the resolution profile of yeast cells is also variable when grown in various media, which may also influence the repeatability of an assay. Additionally, different amino nitrogen ratios in the various growth media used in the present study were suspected to alter the protein content in the cell wall of yeast, which consequently influenced the efficiency of dye staining and cytometric detection. Thus, the results of the present study provide novel technical information to flow cytometry research and should be taken into consideration in the experimental design of future cell cycle studies involving yeast or other fungi.

## Conflict of interest

The authors declare no conflict of interest.

## Author contributions

ZG conceived and designed the project, performed the experiments, acquired the data, analyzed and interpreted the data, and wrote the paper. MW took responsible for some important steps of the experiments. ZG, MW, and FW discussed the manuscript. Some funds were provided by FW for supporting this project. All the authors read and approved the final manuscript.

## Supporting information


**Fig. S1.** Non‐notable refinement of cytometric resolution by sonication and invisible autofluorescence baseline of yeast cells. (A–C). Histograms of yeast cells growing in the minimal medium. (D–F). Histograms of yeast cells growing in SD medium. (G–I). Histograms of yeast cells growing in DTB medium. (J–L). Histograms of yeast cells growing in YPD medium. (A, D, G, J). Histograms of sonicated yeast samples. (B, E, H, K). Histograms of non‐sonicated samples. (C, F, I, J). Histograms of non‐stained samples. The above results indicated that, compared to the non‐sonicated samples, sonication had a minimal contribution to the refinement of the resolution, which is now regarded as unnecessary for the cytometry assays. Moreover, the cytometry of the non‐stained cells confirmed an invisible autofluorescence baseline, indicating that yeast cells had little autofluorescence. The staining protocol for cells in the minimal medium is shown in Fig. 1A15,B15,C15. The staining protocol for cells in SD medium is shown in Fig. 2A15,B15,C15. The staining protocol for cells in DTB medium is shown in Fig. 3A15,B15,C15. The staining protocol for cells in YPD medium is shown in Fig. 4A15,B15,C15.
**Table S1.** Ingredients, amino nitrogen ratio, and total nitrogen ratio of the minimal medium.
**Table S2.** Ingredients, amino nitrogen ratio, and total nitrogen ratio of SD medium.
**Table S3.** Ingredients, amino nitrogen, and total nitrogen of DTB medium.
**Table S4.** Ingredients, amino nitrogen, and total nitrogen of YPD medium.

## Data Availability

The raw data were uploaded to FlowRepository (access number: FR‐FCM‐Z57C).
