# Supplementary material for: Flow cytometric resolution of yeast is affected by enzymatic treatment and culture media
Source: FEBS Open Bio. 2022 Aug 2;12(9):1623–33. doi: 10.1002/2211-5463.13456 (PMC9433820; doi:10.1002/2211-5463.13456)
Supplement: Supplementary file 1 — Fig. S1. Non‐notable refinement of cytometric resolution by sonication and invisible autofluorescence baseline of yeast cells. (A–C). Histograms of yeast cells growing in the minimal medium. (D–F). Histograms of yeast cells growing in SD medium. (G–I). Histograms of yeast cells growing in DTB medium. (J–L). Histograms of yeast cells growing in YPD medium. (A, D, G, J). Histograms of sonicated yeast samples. (B, E, H, K). Histograms of non‐sonicated samples. (C, F, I, J). Histograms of non‐stained samples. The above results indicated that, compared to the non‐sonicated samples, sonication had a minimal contribution to the refinement of the resolution, which is now regarded as unnecessary for the cytometry assays. Moreover, the cytometry of the non‐stained cells confirmed an invisible autofluorescence baseline, indicating that yeast cells had little autofluorescence. The staining protocol for cells in the minimal medium is shown in Fig. 1A15,B15,C15. The staining protocol for cells in SD medium is shown in Fig. 2A15,B15,C15. The staining protocol for cells in DTB medium is shown in Fig. 3A15,B15,C15. The staining protocol for cells in YPD medium is shown in Fig. 4A15,B15,C15. Table S1. Ingredients, amino nitrogen ratio, and total nitrogen ratio of the minimal medium. Table S2. Ingredients, amino nitrogen ratio, and total nitrogen ratio of SD medium. Table S3. Ingredients, amino nitrogen, and total nitrogen of DTB medium. Table S4. Ingredients, amino nitrogen, and total nitrogen of YPD medium. [file FEB4-12-1623-s001.docx]

Supplementary

**Supplementary Figure 1.** Improvement of cytometry by sonication and autofluorescence situation of yeast cells. **(A-C).** Yeast cells growing in the minimal medium. **(D–F).** Yeast cells growing in SD medium. **(G–I).** Yeast cells growing in DTB medium. **(J–L).** Yeast cells growing in YPD medium. **(A, D, G, J).** Sonicated yeast samples. **(B, E, H, K).** Non-sonicated samples. **(C, F, I, J).** Non-stained samples. The results above have indicated that, as compared with the non-sonicated samples, sonication had a minimal contribution to the improvement of cytometry, which is now regarded as unnecessary for the cytometry assays. Moreover, cytometry of the non-stained cells confirmed the low fluorescence baseline of yeast cells, indicating that yeast cells had little autofluorescence. The staining protocol for cells in the minimal medium is in Figures 1 (A15, B15, C15). The staining protocol for cells in SD medium is in Figures 2 (A15, B15, C15). The staining protocol for cells in DTB medium is in Figures 3 (A15, B15, C15). The staining protocol for cells in YPD medium is in Figures 4 (A15, B15, C15).

**Supplementary Table 1.** Ingredients, amino nitrogen ratio, and total nitrogen ratio of the minimal medium

| Nutrient | Sigma Cat. No. | Molecular weight | Final concentration g/L | Amino nitrogen g/L | Total nitrogen g/L |
| --- | --- | --- | --- | --- | --- |
| L-histidine | H6034 | 155.15 | 0.04 | 0.0012 | 0.0012 |
| L-leucine | L8000 | 131.17 | 0.27 | 0.0291 | 0.0291 |
| L-methionine | M9625 | 149.21 | 0.04 | 0.0043 | 0.0043 |
| L-uracil | U0750 | 112.09 | 0.04 | 0.0114 | 0.0114 |
| Yeast Nitrogen Base [(NH_4_)_2_SO_4_]^*^ | DF0919-15-3^**^ | 132.14^*^ | 5.00^*^ | 0 | 1.0595 |
| Glucose | G8270 | 180.16 | 20.00 | 0 | 0 |
|  |  |  |  |  |  |
| Total |  |  |  | 0.0571 | 1.1055 |

* Yeast nitrogen base (YNB) contains the nitrogen source, ammonium sulfate ((NH_4_)_2_SO_4_). Therefore, the molecular weight of yeast nitrogen base is actually calculated based on ammonium sulfate, which is 132.14. Therefore, the final concentration of YNB is 5.00 g/L, which is only for the nitrogen calculation and comes from the manufacturer information of YNB from Difco^TM^ (<https://www.fishersci.com/shop/products/bd-difco-dehydrated-culture-media-yeast-nitrogen-base-without-amino-acids-6/p-4901538#?keyword=yeast nitrogen base>).

The actual suggested usage of YNB, 6.7 g/L, is in the recipe found in the Cold Spring Harbor Protocols (2015), in the references in this manuscript, and the link below:

<http://cshprotocols.cshlp.org/content/2015/2/pdb.rec085639.short>

The calculations of the concentrations of the amino nitrogen and total nitrogen are also based on the ammonium sulfate in YNB.

** The catalog number of yeast nitrogen base (YNB) of Difco^TM^. The Cold Spring Harbor Protocols offer no suggested manufacture for YNB. The information about the manufacturer of YNB is from the Yeast Protocols Handbook of Clontech^TM^ (Protocol No. PT3024-1, Version No. PR973283), which suggests the product of Difco^TM^ with the catalog number.

The Clontech handbook is found at the link below:

<https://www.takarabio.com/documents/User%20Manual/PT3024/PT3024-1.pdf>

The information about YNB from Difco^TM^ is below:

[https://www.fishersci.com/shop/products/bd-difco-dehydrated-culture-media-yeast-nitrogen-base-without-amino-acids-6/p-4901538#?keyword=yeast nitrogen base](https://www.fishersci.com/shop/products/bd-difco-dehydrated-culture-media-yeast-nitrogen-base-without-amino-acids-6/p-4901538%23?keyword=yeast%20nitrogen%20base)

**Supplementary Table 2.** Ingredients, amino nitrogen ratio, and total nitrogen ratio of SD medium

| Nutrient | Sigma Cat. No. | Molecular weight | Final concentration g/L | Amino nitrogen g/L | Total nitrogen g/L |
| --- | --- | --- | --- | --- | --- |
| L-adenine | A2786 | 135.13 | 0.02 | 0.0104 | 0.0104 |
| L-arginine | A8094 | 174.2 | 0.02 | 0.0064 | 0.0064 |
| L-histidine | H6034 | 155.15 | 0.02 | 0.0054 | 0.0054 |
| L-leucine | L8000 | 131.17 | 0.12 | 0.0128 | 0.0128 |
| L-lysine | L5501 | 146.19 | 0.06 | 0.0115 | 0.0115 |
| L-methionine | M9625 | 149.21 | 0.02 | 0.0019 | 0.0019 |
| L-phenylalanine | P2126 | 165.19 | 0.05 | 0.0042 | 0.0042 |
| L-threonine | T8625 | 119.12 | 0.04 | 0.0047 | 0.0047 |
| L-tryptophan | T0254 | 204.23 | 0.02 | 0.0027 | 0.0027 |
| L-tyrosine | T3754 | 181.19 | 0.02 | 0.0015 | 0.0015 |
| L-uracil | U0750 | 112.09 | 0.02 | 0.0050 | 0.0050 |
| Yeast Nitrogen Base [(NH_4_)_2_SO_4_]^*^ | DF0919-15-3^**^ | 132.14^*^ | 5.00^*^ | 0 | 1.0595 |
| Glucose | G8270 | 180.16 | 20.00 | 0 | 0 |
|  |  |  |  |  |  |
| Total |  |  |  | 0.0666 | 1.1261 |

* Yeast nitrogen base (YNB) contains the nitrogen source, ammonium sulfate ((NH_4_)_2_SO_4_). Therefore, the molecular weight of yeast nitrogen base is calculated based on ammonium sulfate, which is 132.14. Therefore, the final concentration of YNB was 5.00 g/L, which is only for nitrogen calculation and comes from the manufacturer information of YNB from Difco^TM^ (<https://www.fishersci.com/shop/products/bd-difco-dehydrated-culture-media-yeast-nitrogen-base-without-amino-acids-6/p-4901538#?keyword=yeast nitrogen base>).

The actual suggested usage of YNB, 6.7 g/L, is in the recipe in the Cold Spring Harbor Protocols (2015), the references in this manuscript, and the link below:

<http://cshprotocols.cshlp.org/content/2015/2/pdb.rec085639.short>

The calculations of the concentrations of the amino nitrogen and total nitrogen are also based on the ammonium sulfate in YNB.

** The Difco^TM^ catalog number for yeast nitrogen base (YNB). The Cold Spring Harbor Protocols offers no suggested manufacturer for YNB. The information about the manufacturer of YNB is from the Yeast Protocols Handbook of Clontech^TM^ (Protocol No. PT3024-1, Version No. PR973283), which suggests the Difco^TM^ catalog number.

The Clontech handbook is below:

<https://www.takarabio.com/documents/User%20Manual/PT3024/PT3024-1.pdf>

The information about YNB from Difco^TM^ is below:

[https://www.fishersci.com/shop/products/bd-difco-dehydrated-culture-media-yeast-nitrogen-base-without-amino-acids-6/p-4901538#?keyword=yeast nitrogen base](https://www.fishersci.com/shop/products/bd-difco-dehydrated-culture-media-yeast-nitrogen-base-without-amino-acids-6/p-4901538%23?keyword=yeast%20nitrogen%20base)

**Supplementary Table 3.** Ingredients, amino nitrogen, and total nitrogen of DTB medium

| Nutrient | Sigma Cat. No. | Molecular weight | Final Concentration g/L | Amino nitrogen g/L | Total nitrogen g/L |
| --- | --- | --- | --- | --- | --- |
| Dextrose | D9434 | 180.16 | 20.00 | 0 | 0 |
| Tryptone | LP0042^***^ | n/a | 20.00 | 0.7400 | 2.5400 |
|  |  |  |  |  |  |
| Total |  |  |  | 0.7400 | 2.5400 |


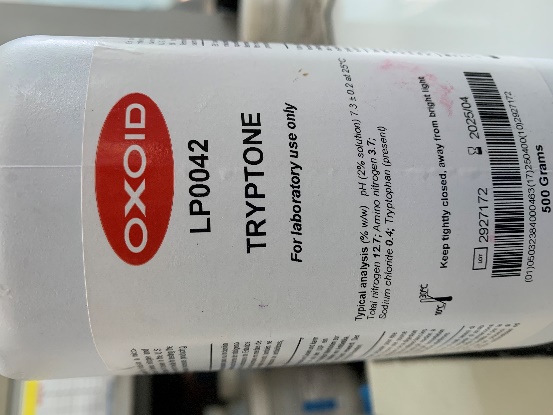
*** The catalog number of tryptone from OXOID-Thermofisher^TM^. The tag of the product provides the information about the concentrations of the amino nitrogen and total nitrogen. (See the photo below)

**Supplementary Table 4.** Ingredients, amino nitrogen, and total nitrogen of YPD medium

| Nutrient | Sigma Cat. No. | Molecular weight | Final Concentration g/L | Amino nitrogen g/L | Total nitrogen g/L |
| --- | --- | --- | --- | --- | --- |
| Glucose | G8270 | 180.16 | 20.00 | 0 | 0 |
| Tryptone | LP0042^***^ | n/a | 20.00 | 0.7400 | 2.5400 |
| Yeast extract | LP0021^****^ | n/a | 10.00 | 0.5100 | 1.1250 |
|  |  |  |  |  |  |
| Total |  |  |  | 1.2500 | 3.6650 |

*** The catalog number of tryptone from OXOID-Thermofisher^TM^. The corresponding information has been provided in Supplementary Table 3.


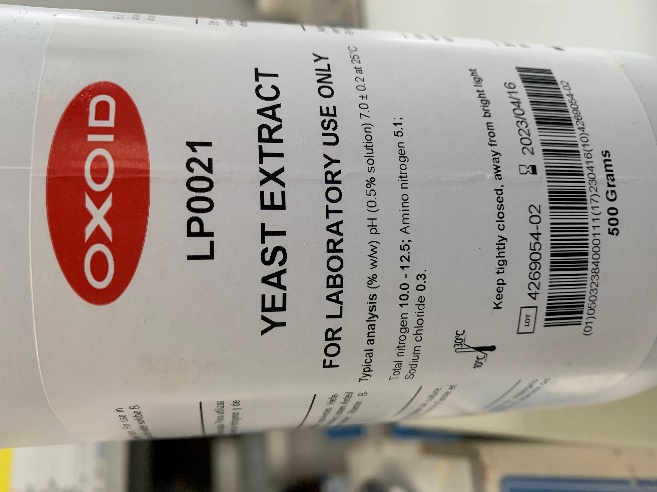
**** The catalog number of yeast extract from OXOID-Thermofisher^TM^. The tag of the product provides the information about the ratios of the amino nitrogen and total nitrogen (see the photo below). The amino nitrogen concentration is 5.1% (w/w), but the total nitrogen concentration is 10.0–12.5% (w/w). Therefore, the total nitrogen concentration was determined to be 11.25% (w/w), which is the mean of the two numbers above.
